# Gene Disruption of Honey Bee Trypanosomatid Parasite, *Lotmaria passim*, by CRISPR/Cas9 System

**DOI:** 10.3389/fcimb.2019.00126

**Published:** 2019-04-26

**Authors:** Qiushi Liu, Jing Lei, Tatsuhiko Kadowaki

**Affiliations:** Department of Biological Sciences, Xi'an Jiaotong-Liverpool University, Suzhou, China

**Keywords:** honey bee, trypanosomatid, *Lotmaria passim*, CRISPR/Cas9, genome editing

## Abstract

Two trypanosomatid species, *Lotmaria passim* and *Crithidia mellificae*, have been shown to parasitize honey bees to date. *L. passim* appears to be more prevalent than *C. mellificae* and specifically infects the honey bee hindgut. Although the genomic DNA has been sequenced, the effects of infection on honey bee health and colony are poorly understood. To identify the genes that are important for infecting honey bees and to understand their functions, we applied the CRISPR/Cas9 system to establish a method to manipulate *L. passim* genes. By electroporation of plasmid DNA and subsequent selection by drug, we first established an *L. passim* clone expressing tdTomato or Cas9. We also successfully disrupted the endogenous *miltefosine transporter* and *tyrosine aminotransferase* genes by replacement with drug (hygromycin) resistant gene using the CRISPR/Cas9-induced homology-directed repair pathway. The *L. passim* clone expressing fluorescent marker, as well as the simple method for editing specific genes, could become useful approaches to understand the underlying mechanisms of honey bee-trypanosomatid parasite interactions.

## Introduction

The honey bee (*Apis mellifera*) plays important roles in agricultural crop production and ecosystem conservation across the globe (Klein et al., 2007; Aizen et al., 2008; Potts et al., 2010). However, a decline in managed honey bee colonies has been observed in North America, Europe, and a part of Asia since 2006. The underlying reasons for the large-scale colony losses are complex, but can be divided into several different categories: inadequate food supplies, anthropogenic chemicals, and exposure to various pathogens/parasites (Goulson et al., 2015). There are diverse honey bee pathogens/parasites, such as viruses, bacteria, fungi, protozoans, and mites (Evans and Schwarz, 2011). Among the protozoans, two *Trypanosomatidae, Lotmaria passim* and *Crithidia mellificae*, were shown to infect honey bees. *C. mellificae* was first identified in Australia in 1967 (Langridge and Mcghee, 1967). Later, in 2015, another novel trypanosomatid parasite infecting honey bees was discovered and named *L. passim* (Schwarz et al., 2015). *L. passim* was found to be more prevalent than *C. mellificae* (Schmid-Hempel and Tognazzo, 2010; Morimoto et al., 2013; Cepero et al., 2014; Ravoet et al., 2014, 2015; Cersini et al., 2015; Schwarz et al., 2015; Arismendi et al., 2016; Cavigli et al., 2016; Stevanovic et al., 2016; Vavilova et al., 2017; Regan et al., 2018) and fewer honey bee colonies were reported to be infected by *C. mellificae* (Ravoet et al., 2015). Thus, *L. passim* rather than *C. mellificae* is likely to be associated with the previously reported winter mortality of honey bee colonies (Ravoet et al., 2013). However, the effects of *L. passim* infection on honey bee health and colony is poorly understood. *L. passim* specifically infects the honey bee hindgut and triggers the expression of antimicrobial peptide (AMP) genes, such as *Defensin 1* and *Abaecin*. In addition, *L. passim* stimulated the expression of genes encoding several downstream components of immune pathways (honey bee orthologs of Imd and Dscam) (Schwarz and Evans, 2013). The genome of *L. passim* was sequenced and appears to be diploid (Runckel et al., 2014) (*L. passim* was referred to *C. mellificae* strain SF in the reference); nevertheless, both *C. mellificae* and *L. passim* have not been fully investigated to date.

*Crithidia bombi*, a trypanosomatid parasite of the bumble bee, has been well characterized. *C. bombi* infection dramatically reduces colony-founding success, male production, and colony size (Brown et al., 2003). Furthermore, *C. bombi* infection was also reported to impair the ability of bumble bees to utilize floral information (Gegear et al., 2006). *C. bombi* infection induces expression of several immune-related genes: *MyD88, Relish, Thioester-containing protein 7* (Schlüns et al., 2010), as well as AMP such as *Abaecin, Defensin*, and *Hymenoptaecin* in bumble bees (Riddell et al., 2011, 2014). The genomes of *C. bombi* and *Crithidia expoeki* were recently sequenced and the sequences revealed signs of concerted evolution of genes potentially important for interaction with the host (Schmid-Hempel et al., 2018).

Recently, a new method based on the CRISPR (clustered regularly interspaced short palindromic repeats)/Cas9 system has become widely used for genome editing (Ran et al., 2013). It has also been applied to edit the genomes of various trypanosomatid parasites: *Trypanosoma cruzi* (Lander et al., 2015, 2016, 2017; Peng et al., 2015), *Trypanosoma brucei* (Beneke et al., 2017; Rico et al., 2018), *Leishmania major* (Sollelis et al., 2015; Beneke et al., 2017), *Leishmania donovani* (Zhang and Matlashewski, 2015; Martel et al., 2017; Zhang et al., 2017), and *Leishmania mexicana* (Beneke et al., 2017). Although the nonhomologous end-joining (NHEJ) pathway appears to be absent in trypanosomatid parasites (Passos-Silva et al., 2010), the endogenous genes were successfully disrupted by the microhomology-mediated end joining (MMEJ) and homology-directed repair (HDR) pathways, in order to repair Cas9-induced double-strand DNA breaks (DSBs). It has been reported that *T. cruzi, T. brucei, L. donovani, L. major* and *L. mexicana* use HDR pathway to repair the DSB when the homologous donor DNA is present (Lander et al., 2015, 2016, 2017; Peng et al., 2015; Sollelis et al., 2015; Zhang and Matlashewski, 2015; Beneke et al., 2017; Chiurillo et al., 2017; Zhang et al., 2017; Rico et al., 2018). Furthermore, *T. cruzi* and *L. donovani* were shown to use MMEJ pathway to repair the DSB in the absence of homologous donor DNA (Peng et al., 2015; Zhang and Matlashewski, 2015).

In this study, we first generated *L. passim* clone expressing fluorescent marker and then attempted to use CRISPR/Cas9 for the genome editing. We will discuss how these approaches can be used to better understand honey bee-trypanosomatid parasite interactions.

## Materials and Methods

### Culture of *L. passim*

*Lotmaria passim* strain SF (PRA-403) was obtained from the American Type Culture Collection (ATCC) and cultured in the modified FP-FB medium (Salathe et al., 2012) at 25°C without CO_2_. To monitor the growth rate of *L. passim*, the parasites were first inoculated at 5 × 10^5^/mL and their number during the culture was measured by CASY® Cell Counter together with Analyzer System Model TT (OMNI Life Science).

### Electroporation of *L. passim* Followed by the Single Clone Isolation

Actively growing *L. passim* (10^7^/mL) was collected, washed twice, and resuspended in 0.4 mL of Cytomix buffer without EDTA (20 mM KCl, 0.15 mM CaCl_2_, 10 mM K_2_HPO_4_, 25 mM HEPES, and 5 mM MgCl_2_, pH 7.6) (Van Den Hoff et al., 1992; Ngo et al., 1998). 2 × 10^7^ parasites were electroporated with 10 μg of pTrex-Neo-tdTomato (Canavaci et al., 2010) or pTrex-b-NLS-hSpCas9 (Peng et al., 2015) using a Gene Pulser X cell electroporator (Bio-Rad). For co-transfection of sgRNA expression vector and donor DNA, 10 μg of the plasmid DNA and 25 μg of the linearized donor DNA were electroporated. The parasites and DNA were mixed well and maintained on ice for 15 min in a cuvette (2 mm gap) before electroporation. The voltage, capacitance, and resistance were set at 1.5 kV, 25 μF, and infinity, respectively. The electroporation was repeated twice with interval of 10 s. *L. passim* electroporated with tdTomato- or Cas9-expressing plasmid DNA was immediately cultured in the modified FP-FB medium containing either 50 μg/mL G418 (SIGMA) or 5 μg/mL blasticidin (InvivoGen), respectively. The parasites started growing about 10 days later. Cas9-expressing parasites electroporated with the sgRNA expression vector and donor DNA were cultured for the first 24 h in the medium without drug followed by addition of 50 μg/mL G418, 5 μg/mL blasticidin, and 10 μg/mL hygromycin (SIGMA). Each drug suppressed the growth rate of *L. passim* to 50% at the half of above concentration. For single clone isolation, the electroporated parasites growing in the above medium were diluted and spread on 1 (w/v) % agarose containing the same medium and drug(s). After 10–15 days, the individual colonies were picked and expanded in 12-well plate. We used serial dilution for the single clone isolation to compare the gene disruption by CRISPR/Cas9-induced HDR and homologous recombination.

### Assessing the Efficiency of Transient Transfection of *L. passim* by Electroporation

*Lotmaria passim* was electroporated with pTrex-Neo-tdTomato as described above with either 1.2, 1.5, or 1.8 kV. The electroporated cells were cultured with FP-FB medium for 48 h, and then 30,000 cells were analyzed for the intensity of red fluorescence using FACS. The experiments were repeated three times.

### Western Blot

The parasites expressing Cas9 tagged with FLAG epitope at the N-terminus were directly suspended with a sample buffer [50 mM Tris-HCl, pH 6.8, 2 (w/v) % SDS, 6 % (v/v) Glycerol, 2 mM DTT, 0.01 % (w/v) Bromophenol Blue] for SDS-PAGE and heated at 99°C for 3.5 min. The cell lysates were separated by two 8 (w/v) % SDS-PAGE gels, and then the proteins in one gel were transferred to a PVDF membrane. Another gel was stained by Coomassie Brilliant Blue as the loading control. The membrane was blocked with 5% (w/v) bovine serum albumin (BSA)/TBST [10 mM Tris-HCl, pH 8.0, 150 mM NaCl, 0.1 (v/v) % Tween-20] and then incubated with a rabbit anti-FLAG antibody (SIGMA, 100-fold dilution) at 4°C overnight. After washing the membrane three times with TBST, it was incubated with IRDye® 680 RD anti-rabbit secondary antibody (10, 000-fold dilution) in 5 (w/v) % skim milk/TBST at room temperature for 1 h. The membrane was washed three times for 30 min and then scanned/analyzed by Odyssey® scanner (LI-COR Biosciences).

### Gene Disruption of *L. passim* by CRISPR/Cas9-Induced HDR

*Miltefosine transporter (LpMT)* and *tyrosine aminotransferase (LpTAT)* genes were annotated from *L. passim* genome sequence (Supplementary File 1) and the sgRNA sequences were designed using a custom sgRNA design tool (http://grna.ctegd.uga.edu) (Peng et al., 2015). Two complementary oligonucleotides corresponding to these sgRNA sequences (0.1 nmole each) were phosphorylated by T4 polynucleotide kinase (NEB) followed by annealing (Ran et al., 2013) and cloning into BbsI digested pSPneogRNAH vector (Zhang and Matlashewski, 2015). The donor DNA for *LpTAT* gene was constructed by fusion PCR of three DNA fragments: 5′ (438 bp) and 3′ (500 bp) UTRs of *LpTAT* and the open reading frame (ORF) of *hygromycin B phosphotransferase* gene derived from pCsV1300 (Park et al., 2013). Similarly, the donor DNA for *LpMT* was prepared as above except the 5′ UTR (540 bp) and the part of ORF downstream of the sgRNA target site (500 bp) were used for the fusion PCR. The fusion PCR products were cloned into EcoRV site of pBluescript II SK(+) and the linearized plasmid DNA by HindIII-HF was used for electroporation as described above. After co-transfection, the drugs resistant clones were selected.

### Genomic PCR

Genomic DNA was extracted from the parasites using DNAiso reagent (TAKARA) and PCR was carried out using KOD-FX DNA polymerase (TOYOBO) and the specific primers shown in the Supplementary File 2. The PCR products were gel purified and directly sequenced to verify the amplified DNA sequences.

### T7 Endonuclease I Assay

After transfection of *L. passim* expressing Cas9 with *LpMT* sgRNA expression vector, the parasites were cultured with FP-FB medium containing 100 μg/mL G418 and 5 μg/mL blasticidin for 12 days until the cell density reached to 5 × 10^7^/mL. Genomic DNA was extracted from the pooled parasites as above and used as the template for PCR to amplify 500 bp *LpMT* genomic DNA containing the sgRNA targeted site (40 cycles). PCR product (2 μL) was treated with 1 unit of T7 endonuclease I (NEB) at 37°C for 15 min. Then, the sample was immediately put on ice-water mixture to terminate the reaction and analyzed by electrophoresis with 2 (w/v) % agarose gel (Kondo and Ueda, 2013).

### Detection of *LpMT* and *LpTAT* mRNAs by RT-PCR

Total RNA was extracted from wild type, *LpMT*, and *LpTAT* heterozygous and homozygous mutant parasites using TRIzol reagent (SIGMA) and treated with 1 unit of RNase-free DNase (Promega) at 37°C for 30 min. 0.2 microgram of total RNA was reverse transcribed using ReverTra Ace (TOYOBO) and random primer followed by PCR with KOD-FX DNA polymerase and the gene specific primers listed in Supplementary File 2.

### Assay for Tyrosine Aminotransferase Activity

Ten milliliter cultures of wild type and *LpTAT* mutant parasites at the density of 10^7^/mL were centrifuged at 2,000 × g for 5 min at 4°C. The cell pellet was washed once with phosphate buffered saline (PBS) and resuspended with 0.8 mL PBS (ice cold). The cell suspension was sonicated for 5 s at the amplitude 1 four times with 30 s intervals using Q700 Sonicator (QSonica, LLC. Newtown, USA) on ice-water mixture. After centrifugation at 15,000 × g for 5 min at 4°C, the supernatant was collected as the cell lysate. Tyrosine aminotransferase activity was measured as previously described (Diamondstone, 1966). The reaction mixture contained 6 mM L-tyrosine (Solarbio), 10 mM α-ketoglutarate (SIGMA), 38 μM pyridoxal-5-phosphate (Adamas), and 3.8 mM sodium diethyldithiocarbamate trihydrate (Aladdin) in 0.2 M potassium phosphate buffer, pH 7.3. The enzyme reaction was initiated by adding 0.2 mL of cell lysate at 25°C in a total volume of 3.2 mL. After 10 min, 0.2 mL of 10 M NaOH was added to stop the reaction and convert p-hydroxyphenylpyruvic acid to p-hydroxybenzaldehyde (pHBA). After 30 min at 25°C, pHBA concentration was measured by recording the absorbance at 331 nm using Varioskan^TM^ LUX multimode microplate reader (Thermo Fisher) with pathlength correction. As the control, NaOH was added to the enzyme reaction mixture prior to the cell lysate. The results were exported and analyzed by SkanIt^TM^ 4.1 software (Thermo Fisher). We defined the amount of enzyme producing 1 μmole pHBA/min at 25°C as one unit. The protein concentration of cell lysate was measured by BCA assay kit (Beyotime) using BSA as the standard and the specific activity of enzyme was determined. The experiments were repeated three times and two-tailed Welch's *t*-test was used for the statistical analysis.

### Quantitative PCR (qPCR) to Analyze the Copy Number of pTrex-Neo-tdTomato Plasmid DNA

Genomic PCR was carried out to detect the part of *tdTomato* ORF in wild type *L. passim* and the parasites stably transfected with pTrex-Neo-tdTomato. The internal transcript spacer region 1 of *ribosomal RNA* gene (*LpITS1*) was PCR amplified as the positive control. The above parasites expressing tdTomato were cultured in the medium for 4 weeks with or without G418 and the genomic DNA was isolated after 2 and 4 weeks. The relative copy number of pTrex-Neo-tdTomato plasmid DNA to the internal control, *LpITS2* in each sample was measured by qPCR using a Hieff^TM^ qPCR SYBR® Green Master Mix (Low Rox Plus, Yesen) and ΔCt method. Three independently isolated genomic DNAs were analyzed for each sample and two-tailed Welch's *t*-test was used for the statistical analysis. The primers used for genomic PCR and qPCR are listed in Supplementary File 2.

## Results

### Generation of *L. passim* Expressing tdTomato

**Figure 1:**
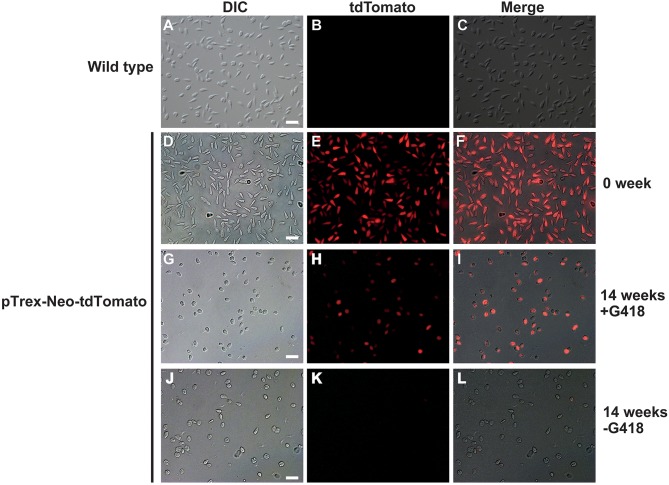
*Lotmaria passim* clone expressing tdTomato. Differential interference contrast (DIC), red fluorescence (tdTomato), and the merged (Merge) images of wild type *L. passim* **(A–C)** and *L. passim* stably transfected with pTrex-Neo-tdTomato. The images of G418-resistant single clone just after the expansion (0 week, **D–F**), cultured for 14 weeks using the modified FP-FB medium with (14 weeks +G418, **G–I**) or without (14 weeks -G418, **J–L**) G418 are shown. White bar = 30 μm.

**Figure 2:**
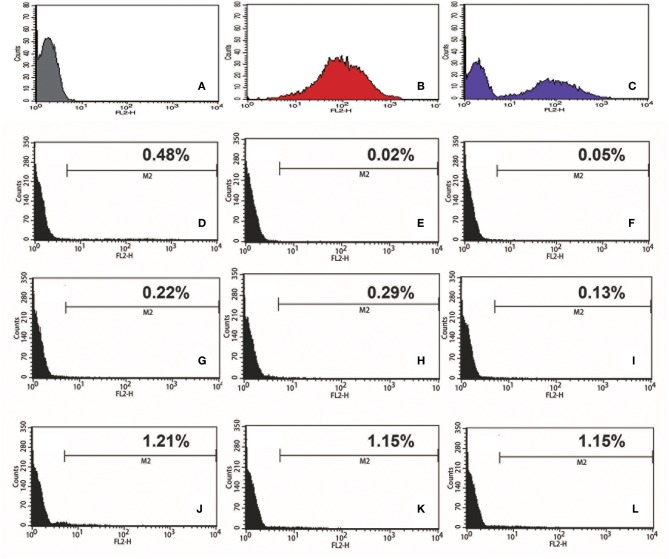
Efficiency of transient transfection of *L. passim* by electroporation. Wild type *L. passim* **(A)**, *L. passim* stably transfected with pTrex-Neo-tdTomato **(B)**, and the mixture of **A** and **B (C)** were analyzed for red fluorescent intensity using FACS. *L. passim* was electroporated with pTrex-Neo-tdTomato at different voltages (**D–F**: 1.2 kV, **G–I**: 1.5 kV, **J–L**: 1.8 kV) followed by culture in the modified FP-FB medium for 48 h. Thirty thousand cells were then analyzed by FACS as above and the experiments were repeated three times. Fraction of tdTomato-expressing parasites is shown as %.

To test if we could generate an *L. passim* clone stably expressing an exogenous protein, *L. passim* was electroporated with plasmid DNA carrying tdTomato and the neomycin resistance gene (*Neo*) driven by the *T. cruzi* rRNA promoter (pTrex-Neo-tdTomato) (Canavaci et al., 2010), followed by G418 selection. Several G418-resistant single clones were isolated from an agarose plate and individually expanded. As shown in Figures 1A–C, wild type *L. passim* was not fluorescent but the parasites stably transfected with pTrex-Neo-tdTomato showed red fluorescence (Figures 1D–F) by expressing tdTomato and the presence of *tdTomato* ORF was also confirmed by genomic PCR (Supplementary File 3); however, most of them lost the expression after 14 weeks in culture (25 passages) without G418 (Figures 1G–L). In fact, the copy number of pTrex-Neo-tdTomato plasmid DNA was reduced in the parasites cultured without G418 after 2 and 4 weeks (Supplementary File 4). These results may suggest that the electroporated plasmid DNA was not integrated into the parasite's chromosomal DNA. The morphology of *L. passim* changed to become more round after long-term culture in the medium with or without G418 (Figures 1A,D,G,J). Consistent with the results of Figure 1, we could distinguish wild type and tdTomato-expressing parasites based on red fluorescent intensity using FACS (Figures 2A–C). Thus, the efficiency of transient transfection of *L. passim* with pTrex-Neo-tdTomato was determined by FACS at 48 h after the electroporation (using 1.2–1.8 kV) (Figures 2D–L). The maximum efficiency was 1.21%, indicating that the selection of stable transfectants by drug is essential.

### Generation of *L. passim* Expressing Cas9

**Figure 3:**
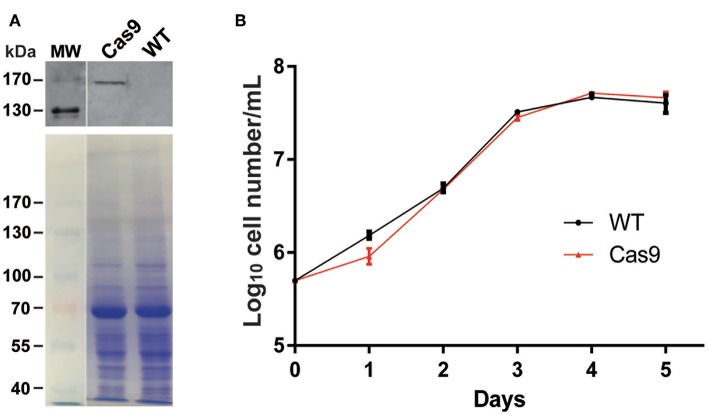
Cas9 protein expression and growth of Cas9-expressing *L. passim* clone. **(A)** The cell lysates of wild type (WT) and FLAG-tagged Cas9 expressing (Cas9) *L. passim* were analyzed by western blot using anti-FLAG antibody (upper panel). The same SDS-PAGE gel was stained by Coomassie Brilliant Blue as the loading control (lower panel). The size (kDa) of protein molecular weight marker (MW) is at the left. **(B)** Growth of WT (black) and Cas9 (red) *L. passim* in the modified FP-FB medium. The experiments were repeated three times and the mean value with error bar (± SD) is indicated for each time point. There is no statistical difference between WT and Cas9 except day 1 (*P* < 0.015) by unpaired *t*-test (two-tail).

We introduced plasmid DNA containing *Cas9* and the blasticidin resistance gene (*Bsd*) driven by the *T. cruzi* rRNA promoter (pTrex-b-NLS-hSpCas9) (Peng et al., 2015) into *L. passim*, followed by blasticidin selection. Cas9 expressed by this plasmid DNA is tagged with FLAG epitope at the N-terminus and was shown to be functional in *T. cruzi* (Peng et al., 2015). Expression of the Cas9 protein was confirmed by western blot (Figure 3A) and growth rate of the Cas9-expressing clone in the presence of blasticidin was comparable to that of the wild type without blasticidin except 1 day after the start of culture (Figure 3B), suggesting that expression of Cas9 does not severely impair growth of *L. passim* in the medium.

### Disruption of Miltefosine Transporter and Tyrosine Aminotransferase Genes in *L. passim* by Cas9-Induced HDR

**Figure 4:**
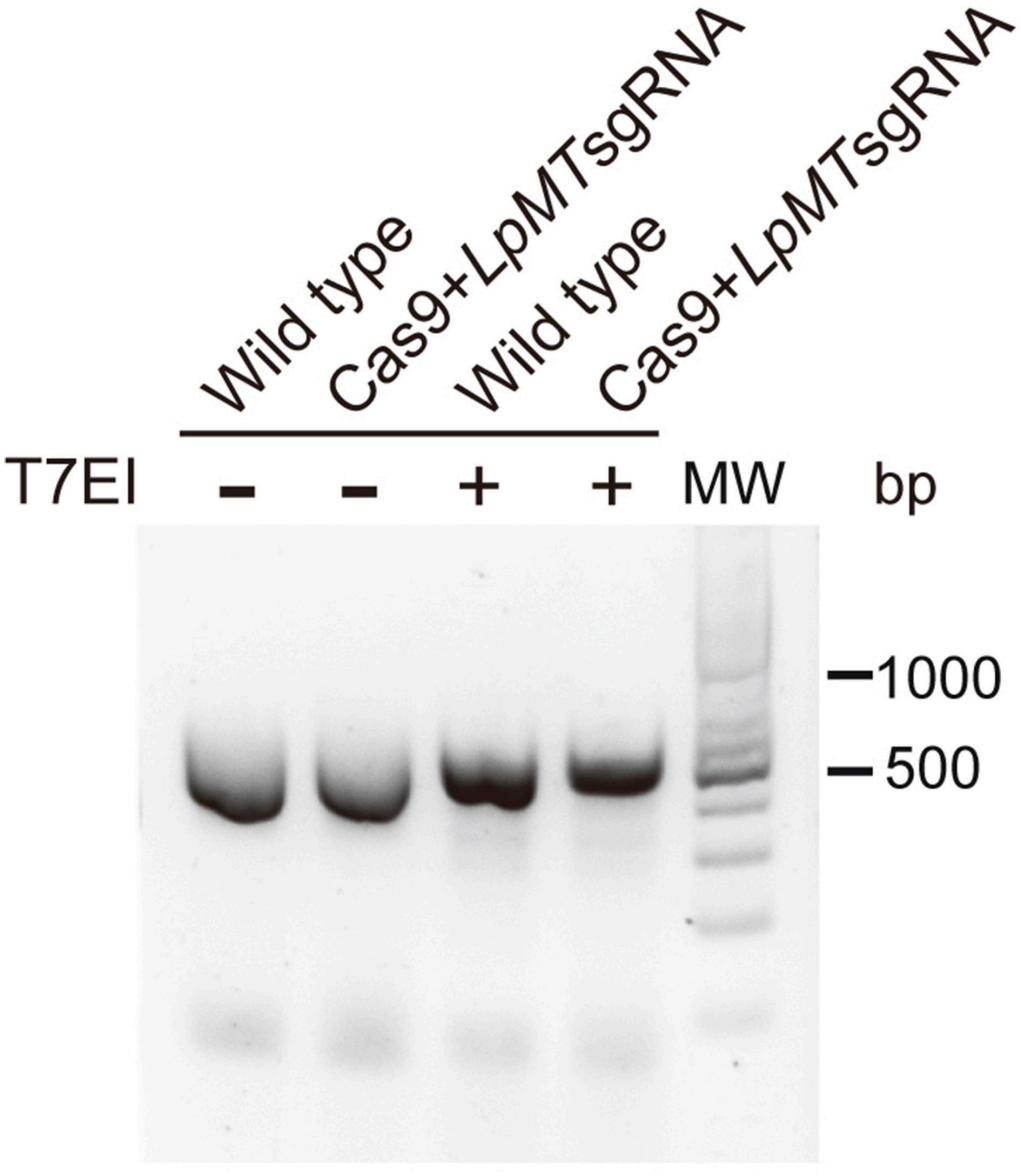
Sensitivity of PCR product amplified with genomic DNA of *L. passim* expressing Cas9 and *LpMT* sgRNA against T7 endonuclease I. Genomic DNA was extracted from wild type *L. passim* and the pooled parasites expressing Cas9 and *LpMT* sgRNA (Cas9+*LpMT* sgRNA) and used as the template to amplify *LpMT* genomic DNA fragment containing the sgRNA targeted site by PCR. The PCR products treated with (+) or without (–) T7 endonuclease I (T7EI) were analyzed by 2% agarose gel. The positions of 500 and 1,000 bp bands in molecular weight marker (MW) are shown at the right.

We first targeted *L. passim miltefosine transporter* (*LpMT*) gene for editing by CRISPR/Cas9 since the homolog was previously disrupted in *L. donovani* (Zhang and Matlashewski, 2015; Zhang et al., 2017). We transfected Cas9-expressing *L. passim* with plasmid DNA that drives the expression of *LpMT*-specific sgRNA and *Neo* under the *L. donovani* rRNA promoter (Zhang and Matlashewski, 2015). *LpMT* genomic DNA fragment containing the sgRNA targeted site was PCR amplified using genomic DNAs extracted from wild type and the pooled blasticidin and G418 resistant parasites. We then tested their sensitivity against T7 endonuclease I digestion. As shown in Figure 4, the PCR products were not digested by T7 endonuclease I, suggesting that DSB induced by the expression of Cas9 and sgRNA appeared to be faithfully repaired without alterations in *LpMT*.

**Figure 5:**
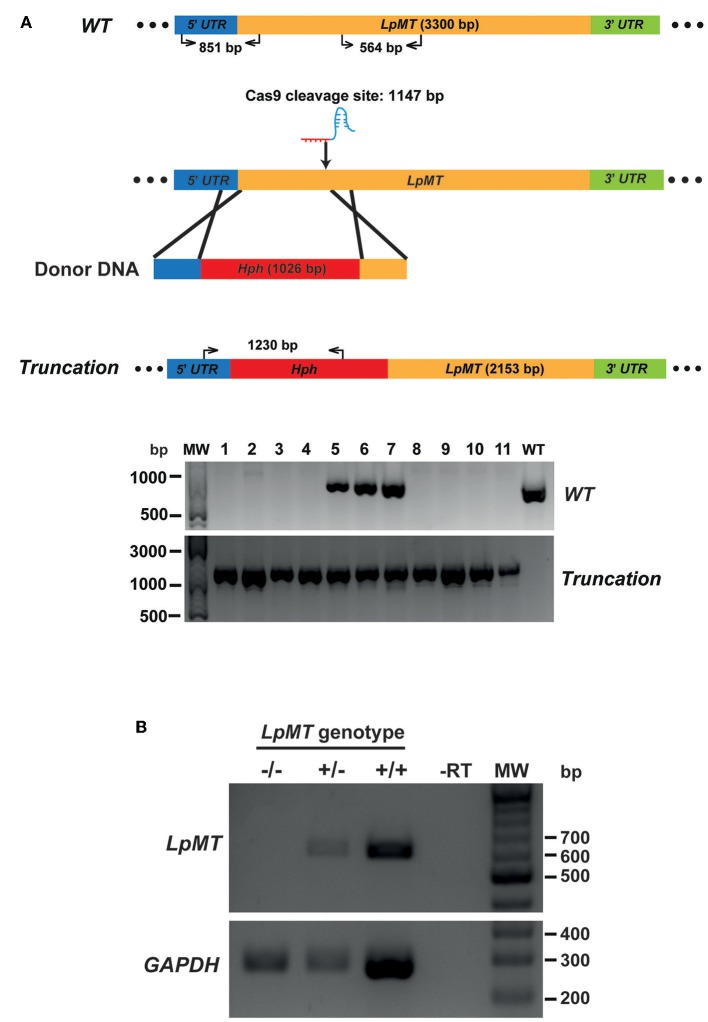
Generation of *miltefosine transporter* disrupted *L. passim* by CRISPR/Cas9-induced homology directed repair. **(A)** Schematic representation of the strategy to truncate *L. passim miltefosine transporter* (*LpMT*) by CRISPR/Cas9-induced homology directed repair (HDR). The positions of primers to detect wild type (*WT*) and truncated (*Truncation*) alleles of *LpMT* are shown with the expected sizes of PCR products. Five prime and 3′ untranslated regions (UTR) as well as open reading frame (ORF) of *LpMT* are shown in blue, green, and yellow, respectively. Donor DNA contains a hygromycin resistance gene (*Hph*, red) flanked by the parts of 5′ UTR and ORF of *LpMT*. The putative cleavage site by Cas9 is at 1,147 bp from the start codon of *LpMT*. Eleven drug (blasticidin, G418, and hygromycin) resistant clones (1–11) together with wild type *L. passim* were analyzed by genomic PCR to detect *WT* (851 bp amplicon) and *Truncation* (1,230 bp amplicon) alleles of *LpMT*. The positions of 500, 1,000, and 3,000 bp bands in molecular weight marker (MW) are shown at the left. **(B)** Detection of *LpMT* and *GAPDH* mRNAs in *LpMT* heterozygous (+/–) and homozygous (–/–) null mutants together with wild type *L. passim* (+/+) by RT-PCR. The expected sizes of RT-PCR products for *LpMT* and *GAPDH* are 564 and 279 bp, respectively. The negative control was run using water as the template (-RT) for RT-PCR. The positions of 200–700 bp bands in molecular weight marker (MW) are shown at the right.

We then transfected Cas9-expressing *L. passim* with both *LpMT*-specific sgRNA expression vector and a donor DNA. The donor DNA contained the hygromycin resistance gene (*Hph)* flanked by 5′UTR (left arm) and a part of ORF downstream of the sgRNA targeting site of *LpMT* (right arm). After the transfection, we selected and expanded the blasticidin-, G418-, and hygromycin-resistant clones. As shown in Figure 5A, all of the drug resistant clones had the *LpMT* truncation allele mediated by *Hph* insertion through HDR; however, three out of 11 clones also contained the wild type allele, suggesting that they were heterozygous. To confirm the truncation of *LpMT*, we examined the mRNA expression in wild type, heterozygous, and homozygous mutant parasites by RT-PCR. *LpMT* mRNA was absent in the homozygous mutant as shown in Figure 5B.

**Figure 6:**
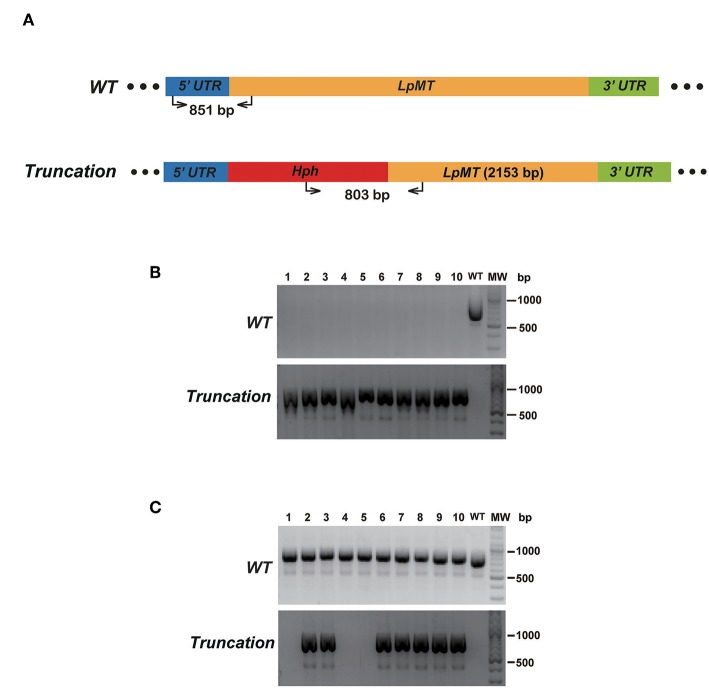
Comparison of disrupting *LpMT* by CRISPR/Cas9-induced HDR and homologous recombination. **(A)** Wild type (*WT*) and truncated (*Truncation*) alleles of *LpMT* are shown as in Figure 5A. The positions of primers to detect *LpMT* truncated allele are shown with the expected size of PCR product. **(B)** Ten drug (blasticidin, G418, and hygromycin) resistant clones (1–10) isolated by CRISPR/Cas9-induced HDR together with wild type *L. passim* were analyzed by genomic PCR to detect *WT* (851 bp amplicon) and *Truncation* (803 bp amplicon) alleles of *LpMT*. The positions of 500 and 1,000 bp bands in molecular weight marker (MW) are shown at the right. **(C)** Ten hygromycin resistant clones (1–10) isolated by homologous recombination were analyzed as in **(B)**.

To verify that replacing the endogenous gene with the donor DNA containing *Hph* was mediated by DNA break-induced HDR rather than homologous recombination of intact genomic DNA, we repeated the truncation of *LpMT* as described above, together with transfecting wild type *L. passim* with the donor DNA only. After culturing the parasites with the drug-containing medium for 61 days, 10 individual drug-resistant clones were isolated, expanded, and analyzed by genomic PCR. Figure 6 shows that all of the 10 clones subjected to CRISPR/Cas9-induced HDR were homozygous truncations; however, all of the 10 clones subjected to the homologous recombination retained the wild type *LpMT* allele, demonstrating that they were heterozygous mutants. Furthermore, 803 bp PCR amplicon representing *LpMT* truncation allele was absent in the three clones (#1, 4, and 5) subjected to the homologous recombination, suggesting that the donor DNA was integrated into *LpMT* with an unexpected orientation or into another locus in the *L. passim* genome. Thus, CRISPR/Cas9–induced HDR is able to replace two alleles of an endogenous gene in *L. passim* with a single donor DNA containing drug-resistant gene.

**Figure 7:**
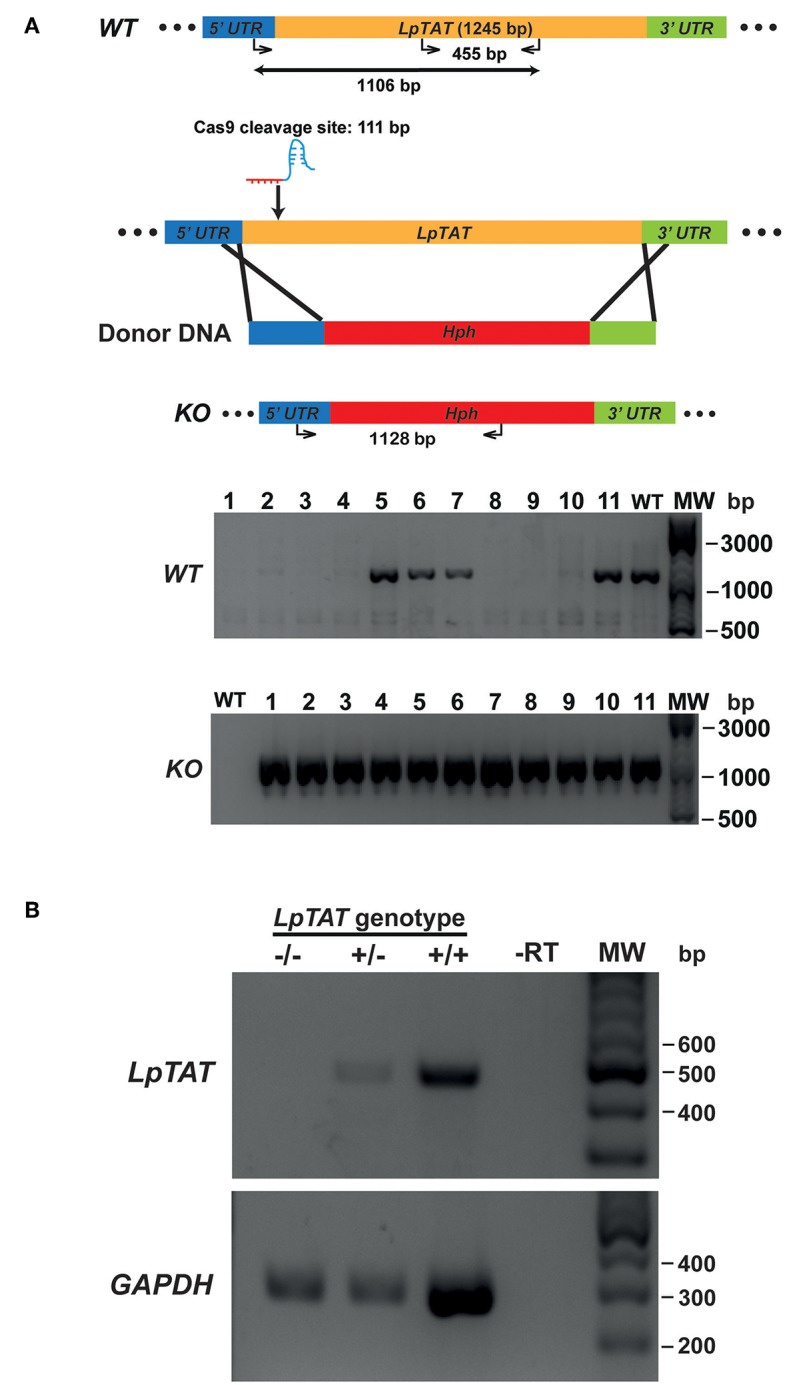
Generation of *tyrosine aminotransferase* disrupted *L. passim* by CRISPR/Cas9-induced HDR. **(A)** Schematic representation of the strategy to disrupt *L. passim tyrosine aminotransferase* (*LpTAT*) by CRISPR/Cas9-induced HDR. Donor DNA contains a *Hph* gene flanked by the parts of 5′ and 3′UTRs of *LpTAT*. The putative cleavage site by Cas9 is at 111 bp from the start codon of *LpTAT*. Eleven drugs (blasticidin, G418, and hygromycin) resistant clones (1–11) together with wild type *L. passim* were analyzed by genomic PCR to detect wild type (*WT*, 1,106 bp amplicon) and knocked out (*KO*, 1,128 bp amplicon) alleles of *LpTAT*. The positions of 500, 1,000, and 3,000 bp bands in molecular weight marker (MW) are shown at the right. **(B)** Detection of *LpTAT* and *GAPDH* mRNAs in *LpTAT* heterozygous (+/–) and homozygous (–/–) null mutants together with wild type *L. passim* (+/+) by RT-PCR. The expected sizes of RT-PCR products for *LpTAT* and *GAPDH* are 455 and 279 bp, respectively. The negative control was run using water as the template (-RT) for RT-PCR. The positions of 200–600 bp bands in molecular weight marker (MW) are shown at the right.

**Figure 8:**
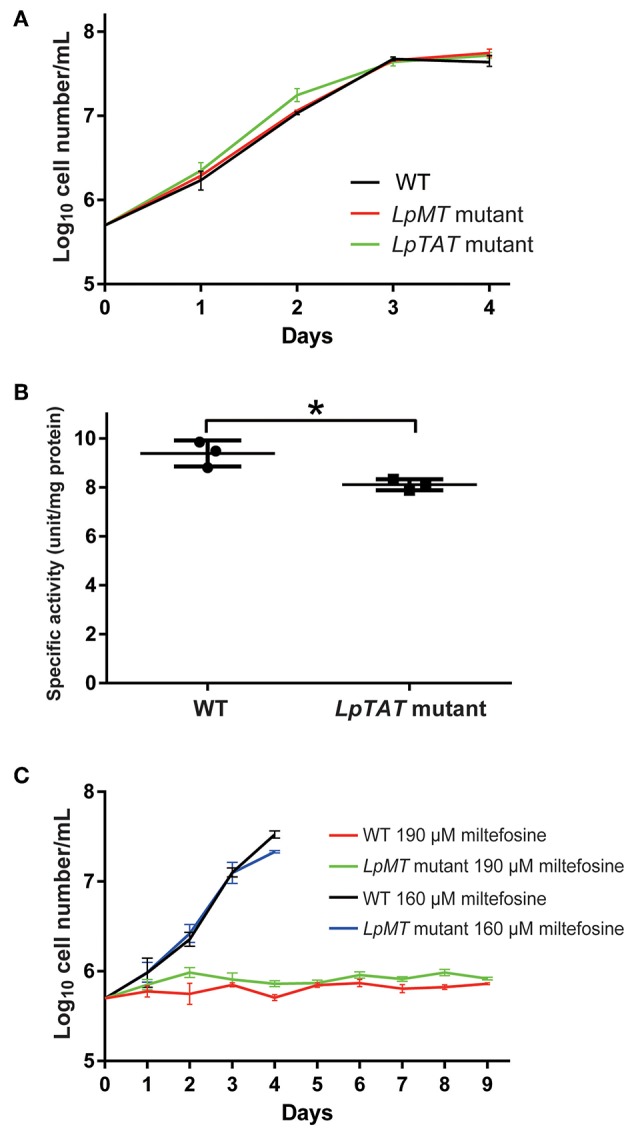
Phenotypic characterization of *LpMT* and *LpTAT* null mutants. **(A)** Growth of *LpMT* (red) *LpTAT* (green) mutants together with wild type (WT, black) in the modified FP-FB medium. The experiments were repeated three times and the mean value with error bar (± SD) is indicated for each time point. There is no statistical difference between WT and two mutants except WT and *LpTAT* mutant at day 2 (*P* < 0.006) by two-tailed Dunnett test. **(B)** Specific activity of tyrosine aminotransferase in wild type (WT) and *LpTAT* mutant. Three protein samples were analyzed for each genotype and asterisk (_*_) indicates the statistical difference between two groups (*P* < 0.02) by one-tailed Welch's *t*-test. **(C)** Growth rates of wild type and *LpMT* mutant in the culture medium with either 160 μM (black and blue) or 190 μM (red and green) miltefosine. The experiments were repeated three times and the mean value with error bar (± SD) is indicated for each time point. In the presence of 160 μM miltefosine, there is no statistical difference between WT and *LpMT* mutant except at day 4 (*P* < 0.02) by two-tailed Welch's *t*-test. With 190 μM miltefosine, WT and *LpMT* mutant are statistically different except at day 1, 3, and 5. *P*-values between them are <0.03, <0.008, <0.05, <0.03, <0.01, and <0.02 at day 2, 4, and 6–9, respectively by two-tailed Welch's *t*-test.

We also successfully disrupted *tyrosine aminotransferase* (*LpTAT*) using Cas9-induced HDR as described above (Figure 7). *LpTAT* is one of *L. passim* genes that become upregulated upon infection of the honey bee hindgut (Liu et al., 2019). As shown in Figure 8A, growth rate of *LpMT* or *LpTAT* null mutant was comparable to that of wild type, suggesting that these two genes are not essential for the parasite's growth in the culture medium. We measured tyrosine aminotransferase activity of wild type and *LpTAT* null mutant and found that wild type has slightly higher specific activity than *LpTAT* mutant (Figure 8B). *L. passim* contains another TAT-like protein sharing 69% identity and 84% similarity with amino acid sequence of LpTAT. These two proteins appear to be functionally redundant and thus disrupting one *LpTAT* gene did not result in the total loss of enzymatic activity. We cultured wild type and *LpMT* null mutant in the medium containing either 160 or 190 μM miltefosine. Growth of wild type and *LpMT* mutant was similar until day 3 with 160 μM miltefosine. In the presence of 190 μM miltefosine, growth of both wild type and *LpMT* mutant was suppressed; however, more viable cells were present with *LpMT* mutant than wild type (for example, 8.3 × 10^5^ ± 1.8 × 10^4^ vs. 7.3 × 10^5^ ± 1.1 × 10^4^ at day 9) (Mean ± SEM, *n* = 3) (Figure 8C). These results demonstrate that disrupting *LpMT* makes *L. passim* only partially resistant to miltefosine as previously observed with *Leishmania* (Pérez-Victoria et al., 2003; Zhang and Matlashewski, 2015).

## Discussion

### Stable Expression of Exogenous Proteins in *L. passim*

We successfully introduced several plasmids to express exogenous proteins (tdTomato and Cas9) in *L. passim*. Although we changed the conditions for electroporation of *L. passim*, the efficiency of transient transfection was quite low (<1.21%), so that the stable transfectants had to be selected by drug(s). Although the introduced plasmid DNA does not appear to integrate into the *L. passim* genomic DNA, it is likely to be present in multicopy, since the parasite has to undergo multiple cell divisions to lose the plasmid DNA. Consistent with the phylogenetic similarity of *L. passim* with other trypanosomatids (Schwarz et al., 2015), the *T. cruzi* and *L. donovani* rRNA promoters are functional for protein and sgRNA expression in *L. passim*. Therefore, various expression plasmids constructed for genome editing of *Trypanosoma* and *Leishmania* by CRISPR/Cas9 could be directly applied to *L. passim* as well. *L. passim* expressing a fluorescent protein, such as tdTomato, could be useful to monitor how the parasite establishes the infection in the honey bee hindgut.

### Gene Disruption in *L. passim* by CRISPR/Cas9-Induced HDR

We were able to successfully disrupt two endogenous genes of *L. passim* by CRISPR/Cas9-induced HDR. In contrast to disrupting a specific gene by homologous recombination, single transfection with sgRNA-expressing plasmid DNA and donor DNA containing a drug-resistant gene was sufficient. However, it usually takes more than 60 days to obtain the homozygous mutant by selecting the drug-resistant parasites in the culture medium followed by isolating the single clones. Considering that we obtained both heterozygous and homozygous mutants for *LpMT* and *LpTAT* (Figures 5, 7), as well as the low transfection efficiency of *L. passim*, the replacement with the donor DNA may occur initially with one allele. CRISPR/Cas9-induced HDR then follows with the second allele, where the first replaced allele serves as the template. This mechanism is similar to the “mutagenic chain reaction” used for converting heterozygous to homozygous mutation in fruit fly (Gantz and Bier, 2015). However, it is also possible that a few homozygous mutants generated immediately after the transfection were primarily expanded during the drug selection. To shorten the selection period, electroporation of heterozygous mutant parasites with donor DNA containing different drug-resistance gene followed by selection using two drugs should be considered. However, if the target gene is essential for survival of *L. passim* and haplosufficient, only heterozygous mutant clones will be selected.

As previously reported (Passos-Silva et al., 2010), NHEJ pathway is absent in trypanosomatids; however, MMEJ pathway is apparently present in *L. donovani* and *T. cruzi* based on the successful gene modifications by expressing Cas9 and sgRNA (Peng et al., 2015; Zhang and Matlashewski, 2015). In *L. passim*, both NHEJ and MMEJ pathways might not exist, perhaps because the parasite lacks the essential genes. Alternatively, Cas9 introduces only a single-strand break, but not DSBs in *L. passim* genomic DNA, so that only the HDR pathway is induced as a result.

We could apply CRISPR/Cas9-induced HDR to prepare a library of *L. passim* clones in which specific genes are mutated. The genes essential for survival could be identified by the absence of homozygous null mutants, and the genes necessary for optimal growth in the culture medium could also be tested. More importantly, we identified *L. passim* mRNAs up- or down-regulated during the infection of honey bee hindgut; for example, *LpGP63* mRNA is continuously up-regulated (Liu et al., 2019). We could therefore establish *LpGP63* mutant by CRISPR/Cas9, and then test the phenotypes by infecting honey bees. If *LpGP63* has the important roles for establishing and maintaining the infection in the honey bee hindgut, we could expect the number of *LpGP63* mutant does not increase compared to wild type. Understanding *L. passim* gene functions by CRISPR/Cas9 will provide insights into the molecular and cellular mechanisms of host (honey bee)-parasite (*L. passim*) interactions.

## Author Contributions

QL and JL conducted all experiments. TK supervised the research project.

### Conflict of Interest Statement

The authors declare that the research was conducted in the absence of any commercial or financial relationships that could be construed as a potential conflict of interest.
