# Supplementary material for: Gene Disruption of Honey Bee Trypanosomatid Parasite, Lotmaria passim, by CRISPR/Cas9 System
Source: Front Cell Infect Microbiol. 2019 Apr 26;9:126. doi: 10.3389/fcimb.2019.00126 (PMC6497781; doi:10.3389/fcimb.2019.00126)
Supplement: Supplementary file 1 [file Table_1.docx]

**Supplementary file 1. Annotated sequences of *L. passim* miltefosine transporter and tyrosine aminotransferase genes**

>*L. passim* miltefosine transporter (*LpMT*)

ATGTCGGGGCGGCAGCCAACGTGGCGGAAGTACTTCCCGTCCAGCGTGTTCCCCGACAAGTGCTGTGACGTCTTGTGCTGCGGCCGCCTTAGCCAAGAGGCGGAGGAAGACGTCGAGGCGGAGGTCATCGTTCACATGAACGATCCGGTGGCCAACGCGGAGTTCAAATACCCGTCCAACTTCATCTGCACCTCCAAGTACACCATGTGGTCCTTCCTCCCCTTGGGCCTCTTCTTTCAGTTCATGAAGGTGAGCAACATCTACTTTTTAATCAATATGGTCTTCAGCCTCATTCCAGGAGTTTCACCCGTGAGCCCAGCCACCGCCGTAGCCCCGCTGGTGGTCGTGGTCGTGGTCGCGCTGATCAAGGAAGGCATTGAGGATATTCGGCGTCACCAGGCGGACAATAAGGCGAACTCCATCGCGGCAATGGTCGTGCGAGGCGACGAGCTCGTCGCGGTCGCAAGCAAAGATGTACAGGCCGGCGACGTGATGTACATCAAAATCGGGGAGGAGGTGCGTGCGGATGTCGTACTCTTCTCCACCTCCGTGGACGAGGGCCAGGCCTTCATCGACACCTGCAACCTCGATGGCGAGACAAGCCTGAAGAGCCGTAAGGCGCTCGAGCACACGTGGTCGCTGAACAGCGTGGAGGCAGTGAAAAATAGCACAGGCGTGCTGCACACGAGCATGCCTGACCCCGGGCTGCTGTCGTGGAACGGCATGTTAGAGCTGAACGGTGAGGAGTTGGCGCTTTCGCTCGACCAGTTTCTCTACCGCGGCTGCATCCTGCGGAACACCGACTGGATCTGGGGCATGGTCGCCTACGCCGGCATCGACACGAAGATGTTCCGCAACTTGAAGGAGAAGCCGCCCAAGTCGTCCAACCTCGACCGCAAGCTAAACTACCTCATCGTCGCCATCTTCATCTTTCAGAACATTATGCTCTTTATTATAGCCTCGCTGGCCGTCTGGTGGAATCACAAGCACCGCGACGCCGTCTACCTCGACTACTTTCTGAAGCAGTACGAGAACGGTCGCCTGTGGGGCTACCGCTACCTTGCCTACTTCATCCTGCTCAGCTACTGCGTGCCCATCTCCCTCTTCATCACGACGGAGCTGTGCAAGGTGATTCAGGCGCAGTGGATGCGGGTGGATTGCCACATGATGGAGTACATGTCTGACCGCTGGCGCCACTGCCAGCCGAACACGTCGAACCTAAACGAGCAGCTCGCCATGGTGCGATTTATCTTTAGCGACAAGACGGGCACGCTGACCGAGAACGTCATGAAGTTCAAGCGCGGCGACGCTCTCGGCTTCCCGATCGACACGAATGACCTGGAGAGCTGCAGGACGCAGATGCGCAAAGAGGAGAGCTCGAGTGGGCTGGGCCCAGTGCAGGAGTACTTCTTAGCCCTCGCCCTGTGTAACACGATCCAGCCCTTCAAGGACGAGGAGCGGGAGCACGGCGTGATTTACGAGGGCAGCTCCCCAGACGAGGTCGCTCTTGTCGAGACGGCCGCGGAGCTTGGGTTCCGCCTGATCAGTCGGACCACCCGCACCATCACCCTGCAGCTGGCGAACGGGACGAAAAAGGTCTACAACGTTCTTGCGACGCTGGAGTTTACGCCGGACCGCAAGATGATGAGCGTCGTTGTCGAGGACAACGACACGAAGCGGGTAACCTTGTACAACAAGGGTGCGGACAGCTTCGTGCGGTCGCAGCTCAGCCGCGGTCCCGACGTGCAGGCGCATATGGAGCGGGTCGACGGCGTGCTGACGGAGATGTCGTCGACGGGGCTGCGCACGTTGCTCGTGTGCGCCAAGGACCTTACCCGCGCTCAATTCGAGACGTGGAATACACGCTTCGTGGAAGCTGGCAAGGTACTCCACAACCGCAGTGAGGAGGTGGACCGGGTGTGCCTGGAGATGGAGAAGGACATGCGCCTCGTCGGTGCCACCGCGATCGAGGACAAGCTGCAGGACCAGGTGCCGGAAACACTCTCCTTTTTCTTAAACGCAGGCGTGGTCGTCTGGATGCTGACGGGCGACAAGCGCGAGACGGCTGTCACGATTGCTACGACGTCGACGCTGTGCGACCCGCGGACGGACTTCGTGGACCACATCGACATCGGCCACTTCGAACCGTCCGCGAGGAGCGCGATCGACAAGGTGGGCCGCGACCTGGAGGTGGTGGAGCAGCACGTCAACTTGAAAGGCAGCGATCAGGAGCGGCGCTGCACCTTCGTCGTCGACGGCCCGGCGCTGAATGTCGCCATGGAGCACTACTTCGAGAAGTTTCTCGCCCTTTCGCAGAAGGTGAACTCCGCCGTCTGCTGCCGCCTCACGCCGATCCAGAAGGCCAACGTGGTGCACATGTTCCAGAAGTCCACCGGGCTGACGGCGCTCGCCATTGGCGACGGCGCCAACGACGTGTCGATGATCCAGGAGGGTCGCGTGGGGATCGGCATCATCGGGCTGGAGGGCGCTCAGGCGGCGCTGGCGGCGGACTACGCCATCCCCCGCTTCAAGCACCTGCGACGTCTCTGCGCCGTACACGGCCGCTACGCCCTCTACCGCAACGCGAGCTGCATCCTCGTGAGCTTCTACAAGAACCTCATCATCGCGGTCTGCCAGTTCATCTTCTCCTTCTTCGTCGGCTTCTCCTCGCAGACGCCGTTTGATGGGTGGGTGCTGACCTTCTTCAACATCGCCCTCACCAGCATCCCGCCCTTCTTCATGGGGATCTTTGACAAGGACCTGCCAGAGGAGGCGCTGCTAGAGCGGCCGAAGCTGTACACGCCGCTCTCGCACGGGGAGTACTTCAACGTGAAGATCCAGGTGCGATGGTTTATTGAGGCGCTCGTCACCGCCGCGGCGGTCTTCTTCATGGCCTACCCGACCATGGTCCACCTCGACGCCTCGAATAGCCGCTACACAGGCAAGCTGAGCGGTACCTTAGTCTATTGCGGCATTCTCACGATCGTCATCACCCGGTTTGCGCTCAACATCCGCTACTGGCAGTGGCTGCAGGCGCTCGGGATCGGCCTCTCCTACTTCTTCTTTATGTTGCTCCTCATCCTCTACTCCGCCATCCCGTCTCTCTTTGGTGACACGAGCTTTTACTTCCACGCCTACACGCTCTGGAGCAGCGGCAAGTACTGGTTTTACATGATTTTGTTTCTGGGGACGGAGCTGGTGATTGTTTTGAGCTGTAAGGTGATTCAGAAGTACGCGTTCCCGACTCTGCGTGATGTGGCGGAGCGTCAGTACGCGTTGCAGCACGGCGGCCACATGTAA

>*L. passim* tyrosine aminotransferase (*LpTAT*)

ATGAGCAGCGCTTCACACTTCCCAGAGGTGCAGTCGTCGAAGCACGCACAGCGGACCCTGCAGCCCCTCACTGAGCTGACGGACAAGATGAAGCCGTCGCACAGCACCAAATCCCTGATCAAGCTGTCGATGGGTGACCCGACCGCCGACGGCAACCTGGTCGCGCCGCAAATCCTCGTGGACGAGATGGTGGACATCGTGAAGTCCAAAGACTTCAACGGCTATCCGCCCGTCGCCGGCTACAACGAGGCGCGCCAGGTCGTGGCTGACTACTGGAAGAAGTTTTGTGGCACGCAGGAGCGCAAGGACCAGATCAAGTGGAAGAACGCTCTCCTCACCTCAGGCGGCTCGCACGCGATTGTGCTCGCCATCAGCGCGCTCTGCAACGAGGGCGACAACCTCCTCGTGTGCGCGCCGGCCTTCCCGCACTACAAGACCGTGTGCGACAGCTACGGTGTGGAGTGCCGCTACTTCCTGCTCGACTCCGCCAAAAACTGGGAGGCCGATCTCGACGCGGCGGCCAAGCTGGTGGACAGCAAGACACGCGGCGTCCTTTTCGTGAACCCGTCCAACCCGTGCGGCAGCAACTATAGCCGCAAGCACGTGGGTGAGATCATTGAGTTCTGCGAGAAGTTCAGCCTACCTCTCATCAGCGACGAGATCTACGCCGAGCTCGTCTTCAAGGGCGAGGTGTTCACCTCCATCGCCGACTTCGACACCGACGTGCCGCGTCTCGTACTGGGCGGGTCGGCGAAGCACGCCGTCACCCCGGGCTGGCGCATTGGCTGGCTCATCCTGGTCGACCGCAAGGGCGTGGCAAAGAACTGGATGAACGGCATAGACCGCCTTTCACAGCTGCTGACGGGCTCGAACTCGATTGGGCAGATGTCGCTGGTGCGTGCCCTCACGAAGATTCCGCAGGACCACGTGGACAGTGTGGTTGCGCAGCTGGAGGCGGGTGCGAAGGTATACAACCGCCTGCTGGAGCACGACATCGGCATCACCTTCGATGCGCCACGTGCGTCGATGTTCGTCATGCTGAAGGTCGACCTCAGCTACTTCAAGGACATCGAGACCGACATGGACTTCTACGAGAAGCTGTTGGATGAAGAAAACGTGCAGGTGCTGCCGGGTGAGATCTTCGGCGTGCATGGCTTCGTACGTGCCACGACGTCGCGTCCGGCCGCCATCATCAACGAGGCAGTCGACCGCATCATCGAGTTCTGCCAGCGCCACAAGAAGTAA
