# Supplementary material for: Gene Disruption of Honey Bee Trypanosomatid Parasite, Lotmaria passim, by CRISPR/Cas9 System
Source: Front Cell Infect Microbiol. 2019 Apr 26;9:126. doi: 10.3389/fcimb.2019.00126 (PMC6497781; doi:10.3389/fcimb.2019.00126)
Supplement: Supplementary file 3 [file Table_3.DOCX]

**Supplementary file 3 Detection of *tdTomato* in *L. passim*** **stably transfected with pTrex-Neo-tdTomato**

The part of *tdTomato* ORF (679 bp) was detected by genomic PCR in *L. passim* stably transfected with pTrex-Neo-tdTomato but not wild type parasites. The internal transcript spacer region 1 of *ribosomal RNA* gene (*LpITS1,* 411 bp) was PCR amplified as the positive control.
