# Supplementary material for: Gene Disruption of Honey Bee Trypanosomatid Parasite, Lotmaria passim, by CRISPR/Cas9 System
Source: Front Cell Infect Microbiol. 2019 Apr 26;9:126. doi: 10.3389/fcimb.2019.00126 (PMC6497781; doi:10.3389/fcimb.2019.00126)
Supplement: Supplementary file 4 [file Table_4.DOCX]

**Supplementary file 4 Copy number of pTrex-Neo-tdTomato plasmid DNA in *L. passim* cultured with or without G418**

*L. passim* stably transfected with pTrex-Neo-tdTomato was cultured in the medium with (+) or without (-) G418 for 2 and 4 weeks. The normalized relative copy number of plasmid DNA to the internal transcript spacer region 2 of *ribosomal RNA* gene was determined in each genomic DNA sample by qPCR and ΔCt method. Asterisks (_**_) indicates the statistically significant difference between two groups (*P*-value < 0.01, two-tailed Welch's *t*-test).
